# Differential Production of Cartilage ECM in 3D Agarose Constructs by Equine Articular Cartilage Progenitor Cells and Mesenchymal Stromal Cells

**DOI:** 10.3390/ijms21197071

**Published:** 2020-09-25

**Authors:** Stefanie Schmidt, Florencia Abinzano, Anneloes Mensinga, Jörg Teßmar, Jürgen Groll, Jos Malda, Riccardo Levato, Torsten Blunk

**Affiliations:** 1 Department of Trauma, Hand, Plastic and Reconstructive Surgery, University of Würzburg, Oberdürrbacher Str. 6, 97080 Würzburg, Germany; Schmidt_S5@ukw.de; 2 Department of Orthopedics, University Medical Center Utrecht, 3508 GA Utrecht, The Netherlands; F.Abinzano@umcutrecht.nl (F.A.); A.Mensinga-4@umcutrecht.nl (A.M.); J.Malda@umcutrecht.nl (J.M.); 3 Department for Functional Materials in Medicine and Dentistry and Bavarian Polymer Institute, University of Würzburg, Pleicherwall 2, 97070 Würzburg, Germany; joerg.tessmar@fmz.uni-wuerzburg.de (J.T.); juergen.groll@fmz.uni-wuerzburg.de (J.G.); 4 Department of Clinical Sciences, Faculty of Veterinary Medicine, Utrecht University, 3584 CM Utrecht, The Netherlands

**Keywords:** ACPC, chondroprogenitors, tissue engineering, MSC, agarose, hypoxia, ECM, co-culture, zonal, cartilage

## Abstract

Identification of articular cartilage progenitor cells (ACPCs) has opened up new opportunities for cartilage repair. These cells may be used as alternatives for or in combination with mesenchymal stromal cells (MSCs) in cartilage engineering. However, their potential needs to be further investigated, since only a few studies have compared ACPCs and MSCs when cultured in hydrogels. Therefore, in this study, we compared chondrogenic differentiation of equine ACPCs and MSCs in agarose constructs as monocultures and as zonally layered co-cultures under both normoxic and hypoxic conditions. ACPCs and MSCs exhibited distinctly differential production of the cartilaginous extracellular matrix (ECM). For ACPC constructs, markedly higher glycosaminoglycan (GAG) contents were determined by histological and quantitative biochemical evaluation, both in normoxia and hypoxia. Differential GAG production was also reflected in layered co-culture constructs. For both cell types, similar staining for type II collagen was detected. However, distinctly weaker staining for undesired type I collagen was observed in the ACPC constructs. For ACPCs, only very low alkaline phosphatase (ALP) activity, a marker of terminal differentiation, was determined, in stark contrast to what was found for MSCs. This study underscores the potential of ACPCs as a promising cell source for cartilage engineering.

## 1. Introduction

Articular cartilage has limited self-renewal capacity, and tissue damage often leads to osteoarthritic changes in the joint. Clinical treatments frequently do not result in functional and long-term stable replacement tissue. Instead, the repair tissue is often of a fibrocartilaginous nature and lacks the native-zone-like structure present in hyaline cartilage [1,2].

Tissue engineering is a promising approach to improve current clinical treatments for cartilage damage. Mesenchymal stromal cells (MSCs) are considered as cell source for cartilage treatments because they can be expanded in 2D cell culture without losing the potential to differentiate chondrogenically, in contrast to chondrocytes [3,4,5]. However, MSCs tend to form fibrous tissue and often undergo terminal differentiation and hypertrophy, which in turn can lead to endochondral ossification and decreased cartilage functionality [6,7,8]. A possible alternative cell source for cartilage engineering has been discovered in the superficial layer of articular cartilage, where the so-called articular cartilage progenitor cells (ACPCs) or chondroprogenitors reside [9,10]. These cells show stem cell-like properties and can be extensively expanded in vitro without losing the ability to differentiate into the chondrogenic, adipogenic, and osteogenic lineage [11,12]. They also appear to have high chondrogenic potential but not to cause endochondral ossification [12,13,14]. Therefore, their use in cell-based therapies could be a potential solution for some of the problems that cartilage regeneration therapies have been struggling with in the past [15]. ACPCs have already been tested in animal models and a pilot clinical study in humans has already been performed [12,16,17,18,19]. Nevertheless, the knowledge regarding these promising cells is still limited.

Different cell types can react very differently to changes in their microenvironment, such as changes to the hydrogel materials used as cell carriers, the local oxygen tension, or co-culture with other cell types. However, only few studies have directly compared chondrogenesis of ACPCs and MSCs in hydrogels so far [18,20,21]. In those studies, ACPCs and MSCs have been cultured in gelatin- and hyaluronan-based hydrogels, respectively, either in monocultures or layered co-cultures [18,20,21]. However, the performance of ACPCs in agarose hydrogels, which are widely used in cartilage engineering and represent a standard chondropermissive system [22,23], has not been evaluated yet. Furthermore, insight into the response of ACPCs seeded in hydrogels to different oxygen tensions is limited. As the physiological oxygen level in the native cartilage microenvironment (1–5%) lies below the atmospheric oxygen level, it is important to also study in vitro tissue development under such physiological hypoxic conditions [24,25]. Another important factor for functional articular cartilage is its zonal architecture, which mostly cannot be restored using currently used clinical treatments. In the field of cartilage tissue engineering, several different approaches exist that try to produce cartilage constructs with zones similar to the native structure. Such zones are, for example, defined by the use of different cells (zone-specific chondrocytes, MSCs) [26,27,28] or different material properties (stiffness gradients, different hydrogels) [29,30,31,32]. The combination of ACPCs in a superficial layer and MSCs in a bottom layer has been tested in other hydrogels with encouraging results [18,20,21]. Furthermore, co-culture between MSCs and chondrocytes has been shown to improve chondrogenesis [33,34,35].

Therefore, in this study, we investigated the chondrogenic differentiation of equine ACPCs in agarose hydrogels and compared their performance to that of MSCs. Chondrogenic differentiation was analyzed by quantitative biochemical assays, histology, immunohistochemistry, and RT-qPCR. The performance of ACPCs and MSCs was evaluated under both hypoxic and normoxic conditions in 3D monoculture constructs and in 3D zonally layered co-culture constructs. ACPCs and MSCs exhibited distinctly differential production of ECM components, with markedly higher glycosaminoglycan (GAG) contents in ACPC constructs under all conditions. Differential GAG production was also reflected in layered co-culture constructs. In ACPC constructs, distinctly weaker staining for undesired type I collagen was observed. Alkaline phosphatase (ALP) activity as a marker of terminal differentiation was decreased by hypoxia in MSCs, but had the lowest levels for ACPCs. Taken together, we provide further evidence that ACPCs are a promising alternative cell source for cartilage engineering.

## 2. Results

### 2.1. Glycosaminoglycan Production under Normoxic Conditions

**Figure 1:**
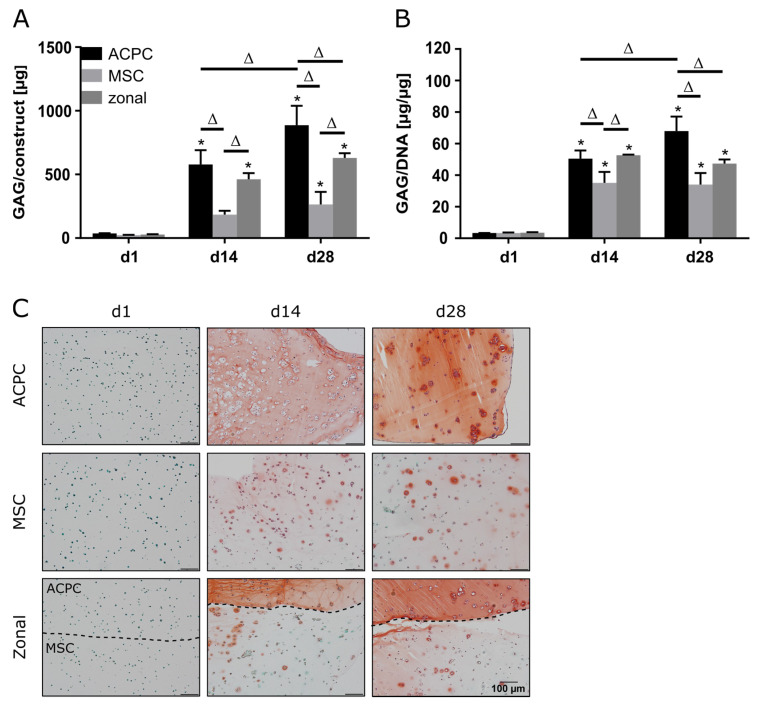
Glycosaminoglycan (GAG) production in articular cartilage progenitor cell (ACPC), mesenchymal stromal cell (MSC), and zonal constructs under normoxic conditions. Biochemical and histological analysis of GAG in agarose hydrogel constructs, seeded with 20.0 × 10^6^ cells mL^−1^, after 1, 14, and 28 days of chondrogenic differentiation under normoxic conditions. ACPCs and MSCs were either cultured alone or in zonally layered co-culture constructs. (**A**) Production of total GAG (GAG/construct) and (**B**) GAG normalized to DNA (GAG/DNA). Data are presented as means ± standard deviations (*n* = 3 biological replicates). Note: (*) indicates statistically significant differences between a day 14 (d14) or day 28 (d28) value and the corresponding day 1 (d1) value of the same group (*p* < 0.05); (Δ) indicates statistically significant differences between groups, or within a group between time points (*p* < 0.05). (**C**) Histological safranin-O staining for visualization of produced GAG. In zonal constructs, the upper layer contained ACPCs and the lower layer contained MSCs (indicated by the dashed line).

ACPCs and MSCs were cultured in 3D agarose hydrogels in chondrogenic medium for 28 days, either alone or in zonally layered co-culture constructs (Supplementary Figure S1). Both ACPCs and MSCs showed production of GAGs in monocultures (non-zonal) and co-cultures (zonal). In gels with ACPCs, distinctly higher amounts of GAGs were detected at day 14 (d14) and 28 (d28) compared to gels with MSCs. Co-culture constructs showed medium values after 28 days (Figure 1A,B). Measurement of the DNA content of different constructs showed higher cell numbers for ACPCs (Supplementary Figure S2); however, GAG production of ACPCs was still higher when normalized to DNA (Figure 1B). Live/dead staining showed similar cell survival in all constructs (Supplementary Figure S3). Histological staining for GAG confirmed the quantitative measurements. In fact, the safranin O staining showed more intense and more evenly distributed signals for ACPCs than for MSCs, which was also reflected in the zonal constructs, with a clearly visible border between layers of ACPCs and MSCs (Figure 1C).

### 2.2. Collagen Production under Normoxic Conditions

**Figure 2:**
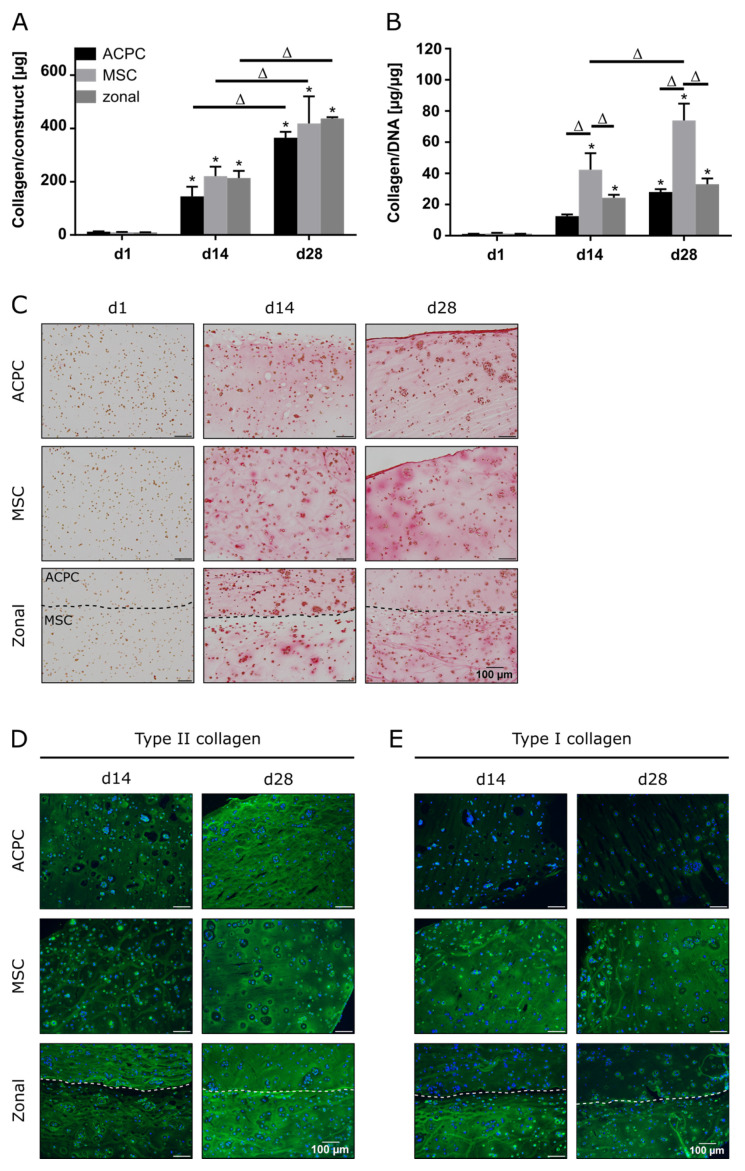
Collagen production in ACPC, MSC, and zonal constructs under normoxic conditions. Biochemical, histological, and immunohistochemical analysis of collagen in agarose hydrogel constructs, seeded with 20.0 × 10^6^ cells mL^−1^, after 1, 14, and 28 days of chondrogenic differentiation under normoxic conditions. ACPCs and MSCs were either cultured alone or in zonally layered co-culture constructs. (**A**) Production of total collagen (collagen/construct) and (**B**) collagen normalized to DNA (collagen/DNA). Data are presented as means ± standard deviations (*n* = 3 biological replicates). Note: (*) indicates statistically significant differences between a d14 or d28 value and the corresponding d1 value of the same group (*p* < 0.05); (Δ) indicates statistically significant differences between groups, or within a group between time points (*p* < 0.05). (**C**) Histological picrosirius red staining for visualization of produced collagen. Immunohistochemical staining for (**D**) type II collagen and (**E**) type I collagen. In zonal constructs, the upper layer contained ACPCs and the lower layer contained MSCs (indicated by the dashed line).

In constructs of all groups, collagen content could be determined, which significantly increased until day 28 (Figure 2A,C). When normalized to the DNA content, it was shown that MSCs produced more collagen than ACPCs (Figure 2B). In immunohistochemical staining of type II collagen, similar intensities for both ACPCs and MSCs were detected (Figure 2D), increasing from day 14 to day 28. In contrast, type I collagen (Figure 2E) was strongly expressed by MSCs, while ACPC constructs showed a distinctly less intense staining at day 14 and day 28. Similar trends were also detected in the zonal constructs, showing similarly intense stainings for type II collagen in both layers at day 28, while at day 14 ACPCs expressed higher levels of type II collagen in co-culture constructs than in monoculture constructs (Figure 2D). For type I collagen, in zonal constructs distinctly stronger staining was detected in the bottom layer with embedded MSCs compared to the ACPC layer. ACPCs showed slightly higher expression of type I collagen in co-culture constructs than in monoculture constructs (Figure 2E). Control stainings of type I and type II collagen at day 1 are shown in Supplementary Figure S4 for comparison. In all the experimental groups, type VI collagen staining (Supplementary Figure S5A) was not spread throughout the whole constructs but was localized in pericellular fashion around the cells, as it is in native articular cartilage, which was observed for all groups.

### 2.3. Glycosaminoglycan Production under Hypoxic Conditions

**Figure 3:**
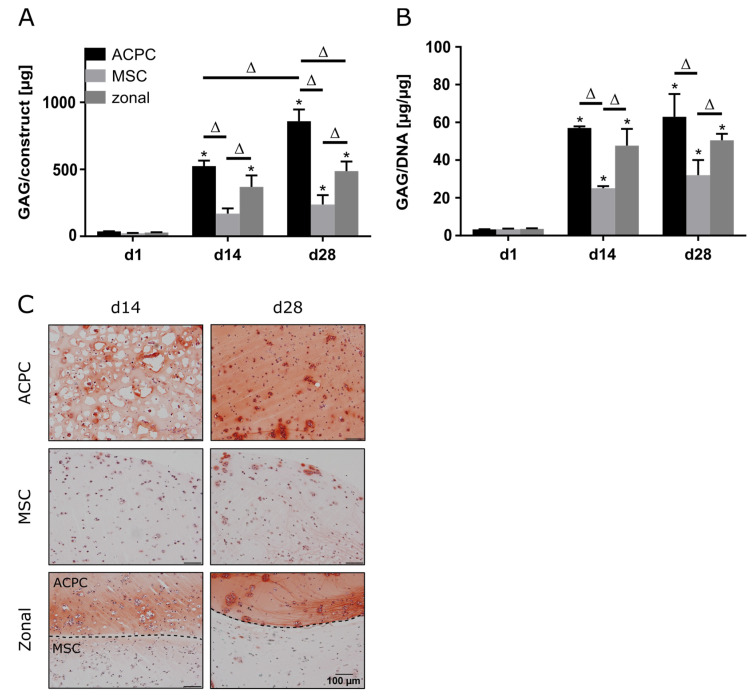
GAG production in ACPC, MSC, and zonal constructs under hypoxic conditions. Biochemical and histological analysis of GAG in agarose hydrogel constructs, seeded with 20.0 × 10^6^ cells mL^−1^, after 1, 14, and 28 days of chondrogenic differentiation under hypoxic conditions. ACPCs and MSCs were either cultured alone or in zonally layered co-culture constructs. (**A**) Production of total GAG (GAG/construct) and (**B**) GAG normalized to DNA (GAG/DNA). Data are presented as means ± standard deviations (*n* = 3 biological replicates). Note: (*) indicates statistically significant differences between a d14 or d28 value and the corresponding d1 value of the same group (*p* < 0.05); (Δ) indicates statistically significant differences between groups, or within a group between time points (*p* < 0.05). (**C**) Histological safranin-O staining for visualization of GAG produced under hypoxic conditions. In zonal constructs, the upper layer contained ACPCs and the lower layer contained MSCs (indicated by the dashed line).

When constructs were cultured at 2% oxygen (hypoxia), ACPCs showed distinctly higher GAG production than MSCs, as observed under normoxic conditions (Figure 3A,B). Similar GAG amounts and GAG/DNA values were determined for normoxic and hypoxic conditions (Figure 1A,B and Figure 3A,B). Again, histological analysis confirmed the quantitative measurements, with more intense staining for ACPCs than for MSCs, which was also mirrored in the zonal constructs (Figure 3C). The effective establishment of an hypoxic environment was verified by assessing the upregulation of key genetic targets associated with hypoxia inducible factors (HIFs), such as phosphoglycerate kinase 1 (*PGK1*), glucose transporter 1 (*GLUT1*), and C–X–C motif chemokine ligand 12 (*CXCL12*), as found in gels cultured under hypoxic in comparison to normoxic conditions (Supplementary Figure S6).

### 2.4. Collagen Production under Hypoxic Conditions

**Figure 4:**
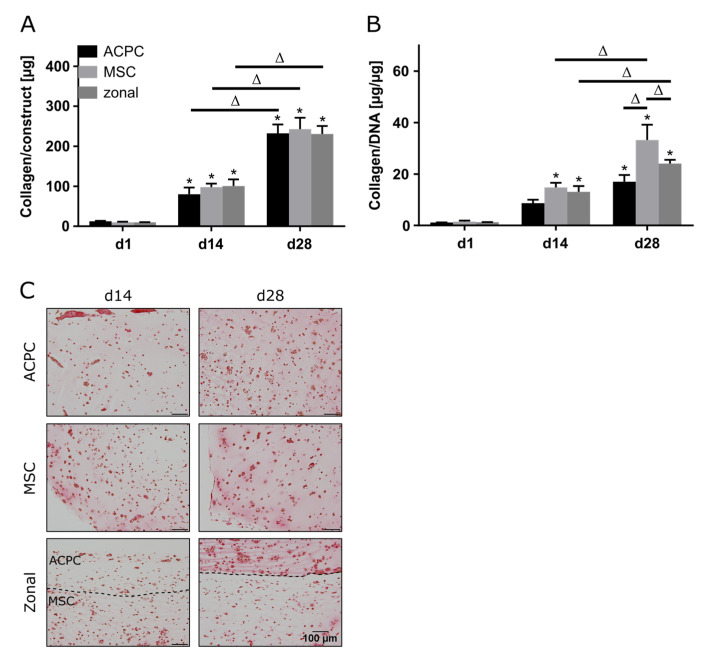

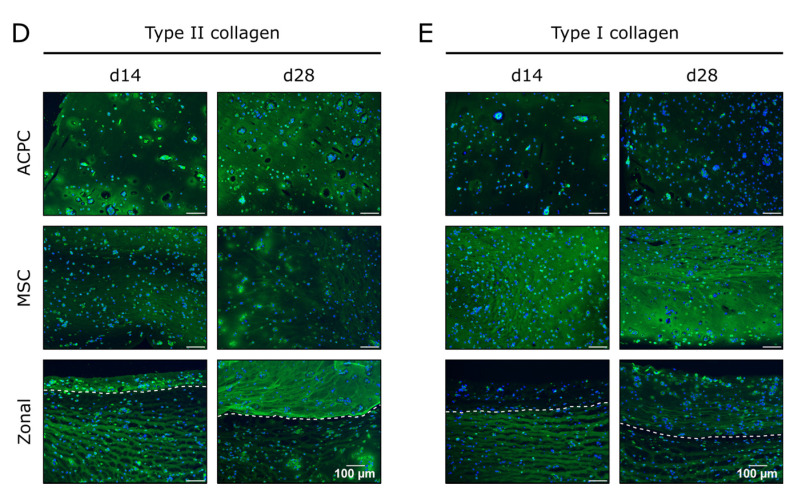
Collagen production in ACPC, MSC, and zonal constructs under hypoxic conditions. Biochemical, histological, and immunohistochemical analysis of collagen in agarose hydrogel constructs, seeded with 20.0 × 10^6^ cells mL^−1^, after 1, 14, and 28 days of chondrogenic differentiation under hypoxic conditions. ACPCs and MSCs were either cultured alone or in zonally layered co-culture constructs. (**A**) Production of total collagen (collagen/construct) and (**B**) collagen normalized to DNA (collagen/DNA). Data are presented as means ± standard deviations (*n* = 3 biological replicates). Note: (*) indicates statistically significant differences between a d14 or d28 value and the corresponding d1 value of the same group (*p* < 0.05); (Δ) indicates statistically significant differences between groups, or within a group between time points (*p* < 0.05). (**C**) Histological picrosirius red staining for visualization of produced collagen under hypoxic conditions. Immunohistochemical staining for (**D**) type II collagen and (**E**) type I collagen under hypoxic conditions. In zonal constructs, the upper layer contained ACPCs and the lower layer contained MSCs (indicated by the dashed line).

Production of collagen was generally lower in hypoxia than in normoxia, but the evolution of collagen synthesis over time showed similar trends for both oxygen pressures. The amounts of total collagen increased significantly over the culture time of 28 days (Figure 4A), and MSCs produced more collagen per cell than ACPCs (Figure 4B). Interestingly, for ACPC constructs, immunohistochemical analysis showed stronger staining for type II collagen (Figure 4D) and distinctly weaker staining for type I collagen (Figure 4E) as compared with MSC constructs. MSC constructs showed distinctly less intense staining for type II collagen in hypoxic (Figure 4D) compared to normoxic conditions (Figure 2D). In zonal co-culture constructs, ACPCs showed more intense staining for type II collagen and MSCs showed less intense staining for type I collagen in comparison to the respective monoculture constructs (Figure 4D,E). The trends of type I and type II collagen were represented in the picrosirius red staining of total collagen (Figure 4C). The staining of type VI collagen, as observed under normoxic conditions, was distributed pericellularly in all groups (Supplementary Figure S5B).

### 2.5. Gene Expression of Proteoglycans and Collagens

**Figure 5:**
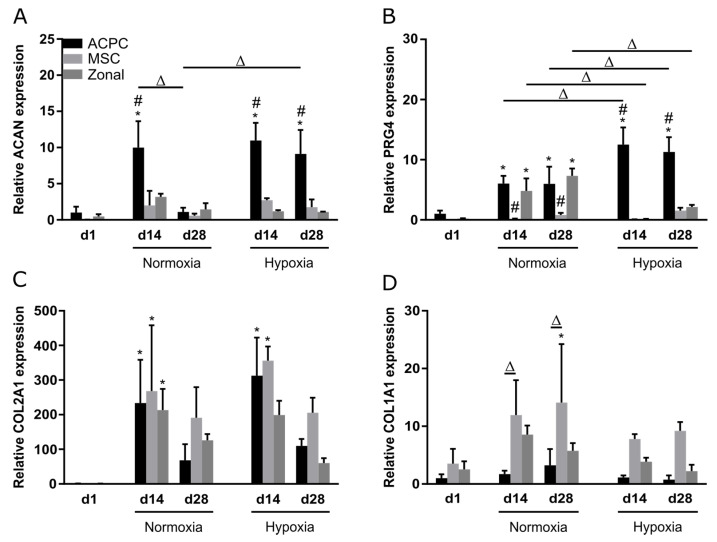
Relative gene expression in ACPC, MSC, and zonal constructs. Gene expression as determined by RT-qPCR in agarose hydrogel constructs, seeded with 20.0 × 10^6^ cells mL^−1^, after 1, 14, and 28 days of chondrogenic differentiation under hypoxic and normoxic conditions. ACPCs and MSCs were either cultured alone or in zonally layered co-culture constructs. Relative expression of (**A**) *ACAN* (encoding aggrecan), (**B**) *PRG4* (lubricin), (**C**) *COL2A1* (type II collagen), and (**D**) *COL1A1* (type I collagen). Data are presented as means ± standard deviations (*n* = 3 biological replicates). Note: (*) indicates statistically significant differences between a d14 or d28 value and the corresponding d1 value of the same group (*p* < 0.05); (#) indicates statistically significant differences of this group compared to the other two groups that share the same time point and oxygen condition (*p* < 0.05); (Δ) indicates statistically significant differences between groups, or within a group between time points (*p* < 0.05).

Relative gene expression of the genes *ACAN, PRG4, COL2A1*, and *COL1A1* was investigated at day 1, day 14, and day 28 under normoxia and hypoxia. Overall, similar observations as in biochemical assays and stainings were made; ACPCs showed distinctly higher expression of proteoglycan genes *ACAN* (Figure 5A) and *PRG4* (Figure 5B) than MSCs, while MSCs expressed higher levels of *COL1A1* (Figure 5D) than ACPCs. Hypoxia and normoxia levels of chondrogenic marker genes *ACAN* (Figure 5A) and *COL2A1* (Figure 5C) did not differ significantly (with exception of the value of *ACAN* expression in ACPCs on day 28 under normoxia). *COL1A1* levels were decreased under hypoxia in all d14 and d28 constructs (Figure 5D). *PRG4* is the gene that encodes lubricin, a typical marker for the superficial zone in cartilage. Under normoxia, *PRG4* expression was similar in zonal constructs and in ACPC constructs. Interestingly, under hypoxia, the expression was downregulated in zonal constructs to low levels similar to MSC constructs.

### 2.6. Activity of Alkaline Phosphatase

**Figure 6:**
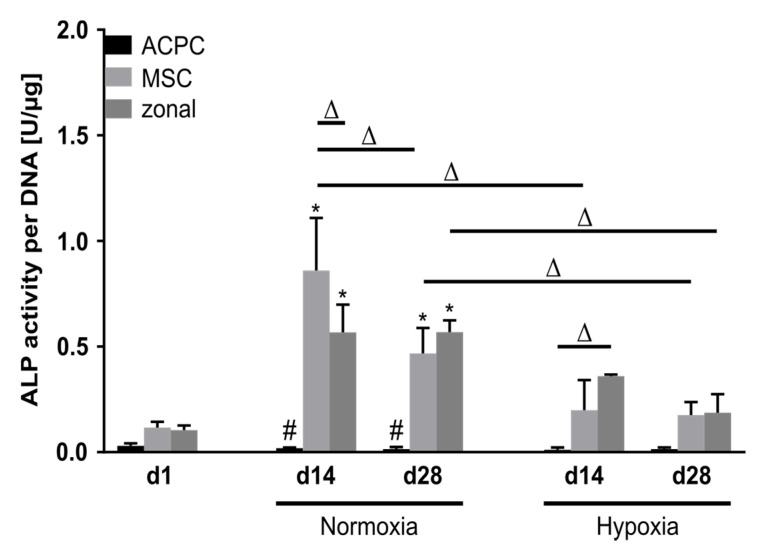
Alkaline phosphatase activity in ACPC, MSC, and zonal constructs. ALP activity in agarose hydrogel constructs, seeded with 20.0 × 10^6^ cells mL^−1^, after 1, 14, and 28 days of chondrogenic differentiation under normoxic and hypoxic conditions. ACPCs and MSCs were either cultured alone or in zonally layered co-culture constructs. Data are presented as means ± standard deviations (*n* = 3 biological replicates). Note: (*) indicates statistically significant differences between a d14 or d28 value and the corresponding d1 value of the same group (*p* < 0.05); (#) indicates statistically significant differences of this group compared to the other two groups that share the same time point and oxygen condition (*p* < 0.05); (Δ) indicates statistically significant differences between groups, or within a group between time points (*p* < 0.05).

Alkaline phosphatase (ALP) activity was analyzed as a marker for terminal differentiation in developing cartilage constructs. At day 1, ALP activity levels were low in all three groups. Remarkably, ACPCs maintained this very low activity level over 28 days in normoxic and hypoxic conditions. In contrast, MSCs and zonal constructs showed elevated ALP activity levels at day 14 and day 28, with significantly reduced values under hypoxia as compared with normoxic conditions (Figure 6).

## 3. Discussion

The choice of cell type is regarded as crucial in cell-based approaches to cartilage regeneration, and in recent years ACPCs have been proposed as a potential new cell source [11,12,13,14,15,16,17,20,21,36]. ACPCs can be expanded in monolayer cultures like MSCs without losing their potential for chondrogenic differentiation, and they might have an advantage over MSCs, as they do not seem to form hypertrophic cartilage [13]. To date, there are few studies exploring the differences between ACPCs and MSCs in hydrogels for cartilage engineering. Therefore, we investigated the chondrogenic differentiation of equine ACPCs and MSCs in agarose hydrogels under normoxic (21%) and hypoxic (2%) conditions, and in monoculture versus zonally layered co-culture constructs.

Both ACPCs and MSCs were able to differentiate chondrogenically in agarose hydrogels and they produced distinct amounts of ECM. However, it also became clear that the cell types specifically synthesized neo-cartilage matrixes with varying compositions. Under normoxic as well as under hypoxic conditions, ACPCs produced markedly higher amounts of GAGs, which were distributed throughout the gels, whereas MSC constructs showed only weak and less homogeneous staining for GAGs. Expression analysis of the genes encoding for the proteoglycans aggrecan and lubricin further supported these findings, with distinctly higher expression in ACPCs as compared with MSCs. Regarding total collagen, MSCs produced higher amounts per cell. Immunohistochemical staining for type II collagen, a marker for hyaline cartilage, appeared similar for ACPCs and MSCs under normoxia and slightly more intense for ACPCs under hypoxia. In contrast, type I collagen clearly was detected at higher levels in MSCs, with only very weak staining in ACPC constructs under both conditions. In this context, it also has to be considered that the DNA content of ACPC constructs was higher than that of MSCs, possibly influencing the staining intensities. Type II collagen expression per cell was, therefore, difficult to compare between MSCs and ACPCs. However, the distinctly less intense staining for type I collagen in ACPC constructs despite the higher cell numbers indicated a distinctly lower type I collagen expression per cell for ACPCs. Type I collagen is normally not present in hyaline cartilage, but is a marker for fibrocartilaginous tissue that is not desirable in articular cartilage regeneration [37]. Fibrocartilage can bring pain relief for the patient, but in the long term it is mostly unable to replace the functions of native articular cartilage [38,39,40]. The distribution of type VI collagen was similar in MSC- and ACPC-derived matrices and reflected its native pericellular localization. Furthermore, high alkaline phosphatase activity, a marker for terminal differentiation, was detected in MSC constructs, while it was hardly detectable at all in ACPC constructs. Taken together, in this study in agarose hydrogels ACPCs clearly outperformed MSCs. They produced a matrix that was more hyaline in comparison to the matrix produced by MSCs; GAG contents were higher in ACPC constructs than in those of MSCs, and collagen production appeared to be qualitatively more suitable in ACPC constructs. Additionally, MSCs showed signs of terminal differentiation, while ACPCs did not.

When looking closely at previous studies that compare the two cell types in other hydrogel systems, two different observations become obvious. In agreement with the present study, in other systems ACPCs were also demonstrated to exhibit a stable phenotype and differentiate chondrogenically without tendencies for hypertrophy or terminal differentiation [12,13,14], while MSCs are known to show signs of hypertrophy and terminal differentiation [6,7,8]. Interestingly, when employing gelMA [20], gelMA/gellan, gelMA/gellan/HAMA [21], and thiol-functionalized hyaluronic acid/allyl-functionalized poly(glycidol) (HA–SH/P(AGE–co–G)) [18] as hydrogels, MSCs produced higher amounts of GAG and collagen than ACPCs (Supplementary Table S1). It should be noted that cells from the same donors under the same cultivation conditions were used in the HA–SH/P(AGE–co–G) study [18] and in the present study with agarose. Thus, the more favorable results for ACPCs as compared to MSCs in the present study clearly underscored the distinct influence of the hydrogel on the cells. More biomimetic hydrogels such as gelMA or hyaluronan-based hydrogels appear to favor MSC differentiation. In contrast, agarose, as used in the present study, has no biological cues, such as integrin-binding domains that MSCs or ACPCs could adhere to, and previous studies that compared MSCs and chondrocytes in agarose or other scaffolds without biological cues [41,42,43] also demonstrated the superiority of chondrocytes over MSCs regarding matrix production. Agarose has been shown to support the native phenotype and morphology of chondrocytes [22,23], and appears to be a suitable environment for chondrocytes and their subpopulation, ACPCs. Taken as a whole, these results support that the selected hydrogel can largely influence the performance of the cells, and the choice of the hydrogel and cell type must be well matched to achieve the desired results [21,44,45,46].

The zonal architecture is regarded as another potentially important factor when considering the development of functional cartilage [47], and mimicking cartilage zones in tissue engineering may be approached by using different cell types or materials for the different layers [20,26,29,48,49]. As it is not possible today to produce cartilage tissue in vitro the same way it develops in the body, these different approaches are employed to recapitulate a zonal architecture that is similar to the native zonal structure and has the potential to mimic some of the properties that render native zonal cartilage more stable than non-zonal repair tissue [48]. In the present study, we used MSCs in a lower layer and ACPCs in an upper layer, because this combination showed promising results in previous studies [18,20]. In monoculture constructs, we observed that ACPCs expressed distinctly higher gene levels of the superficial zone marker *PRG4* than MSCs. This observation was in agreement with previous studies [20,21], and even though we did not analyze the zones of layered constructs separately, this suggested that ACPCs may be a suitable cell source for construction of a superficial zone-like layer in zonal cartilage constructs. In previous studies [18,20,21] employing MSCs in a lower layer and ACPCs in an upper layer, hydrogels were used, in which MSCs produced higher amounts of GAGs and type II collagen than ACPCs. Thus, an ECM distribution similar to the native cartilage structure was approached, with more ECM in the lower layer than in the superficial layer of the hydrogel construct. In contrast, in our agarose constructs, MSCs in the lower layer produced less GAGs than ACPCs in the upper layer; thus, we did not achieve a native-like distribution of ECM in our constructs. However, as co-culture models, the zonal constructs still provided interesting insights into the potential effects of ACPCs and MSCs on each other when these cells are not placed in direct contact and can only communicate through soluble factors. In histological and immunohistochemical stainings, the differences between the zones in layered constructs mostly reflected the differences that could be seen between monoculture ACPC and MSC constructs. However, the intensity of stained type I and type II collagen differed partially between monoculture and co-culture. For example, ACPCs appeared to show stronger staining for type II collagen in co-culture with MSCs (normoxia d14; hypoxia d28), as compared to monoculture; this trend has been observed before in gene expression of type II collagen in an ACPC–MSC zonal co-culture [21]. For type I collagen, co-culture in the presence of ACPCs led to reduced staining in the MSC layer (hypoxia, d14 and d28), again as compared to the respective MSC monoculture. In quantitative biochemical assays for GAG and total collagen, in general the co-culture samples showed values in between those obtained for the ACPC and MSC monocultures, as was also observed in a previous study with cells in separate layers [18]. Furthermore, in the present study co-cultures exhibited distinctly higher levels of ALP activity than ACPCs, similar to MSCs. Previous studies reported that co-culture between MSCs and chondrocytes could improve ECM production and reduce hypertrophy [33,34,35,41,43], however, other studies revealed that these effects required direct contact between the co-cultured cells [50,51]. In our zonal constructs, ACPCs and MSCs had most likely only very limited direct contact with each other, which might be a reason why only comparably small effects of co-culture on ACPCs and MSCs were observed.

Another factor that has been repeatedly reported to enhance chondrogenesis and suppress MSC hypertrophy in tissue engineering is a low oxygen tension, comparable to what chondrocytes experience in their native environment (mostly designated as hypoxia, also called physioxia) [52,53,54,55,56]. However, there have also been studies that did not show improved ECM production under hypoxia [41,57,58]. In our study, in agarose hydrogels, we also detected no significant improvements with regard to ECM production in response to hypoxia. Interestingly, the combination of hypoxia and co-culture with ACPCs reduced type I collagen in MSCs in comparison to monoculture under low oxygen, and thereby directed MSCs towards the production of a more hyaline type of cartilage. Another positive effect of hypoxia was the significant reduction in ALP activity in MSC and co-culture constructs, thereby further increasing the quality of the cartilage tissue formed by MSCs. A downregulation of hypertrophic markers and terminal differentiation of MSCs by hypoxia has also been documented in previous studies [52,55,56,59,60]. In the present study, furthermore, *PRG4* gene expression was significantly upregulated in ACPCs under hypoxia on day 14 and day 28; however, it was significantly downregulated in co-culture constructs under hypoxia on both days. It seemed as if hypoxia enabled MSCs to suppress *PRG4* expression of ACPCs in co-cultures, which might not be a desirable effect, but is still worthwhile investigating in further studies. With regard to ACPCs, only a few studies have investigated chondrogenic differentiation under hypoxia before [25,59]. When cultured in self-organized constructs on fibronectin-coated membranes, human ACPCs, in contrast to articular chondrocytes, showed only minor responses to reduced oxygen tension (5 %) [25]. Similarly, in the present study in agarose hydrogels, only minor effects of hypoxia were detected in equine ACPCs. In future studies, ACPCs may also be investigated further under hypoxia in other hydrogel-based systems.

Some general aspects and limitations regarding the present study may be considered. The study employed agarose hydrogels and was designed as a direct comparison to a study with HA–SH/P(AGE–co–G) hydrogel which investigated constructs in vitro and in an equine in vivo model [18]. In order to facilitate a meaningful comparison, cells from the same two donors (one for each cell type) and the same culture conditions as in the previous study were used. In future studies, further different donors should be included. Nevertheless, the direct comparison of the two studies clearly underscored the possible differential influence of hydrogel materials on the different cell types.

Furthermore, based on previous studies [18,20,21], for ACPCs and MSCs we used different isolation methods, as they were not obtained from the same tissue, and different expansion medium, potentially influencing the ECM production of the cells later. However, the same cells and culture conditions have been used in the study with HA–SH/P(AGE–co–G) hydrogels [18], where in contrast to the present study the MSCs outperformed ACPCs in ECM production. Thus, the comparison strongly suggests that the ECM production in the present study was not a result of MSCs that were compromised or ACPCs that were favored by the conditions of isolation and cultivation. Regarding the zones of layered constructs, we did not analyze the zones separately from each other, but only as a whole construct. For future studies, it would be interesting to analyze separate zones, as well as investigating the exact influence of the cell layers on one another when using other materials. A more general important fact to consider when working with ACPCs is that these cells have to be harvested from cartilage, which may lead to donor site morbidities. However, ACPCs have a high proliferation capacity, and unlike chondrocytes do not lose the ability to differentiate chondrogenically when they are expanded in 2D culture [11,12]. In autologous chondrocyte implantation (ACI), which is routinely conducted in the clinics, chondrocytes are obtained from the rim of the chondral defect; ACPCs could be obtained and expanded from a similar biopsy.

In conclusion, this study demonstrated distinctly differential production of cartilaginous ECM in agarose hydrogels by equine ACPCs and MSCs. For ACPCs, markedly higher GAG content was determined in normoxia and hypoxia, which was also reflected in layered co-culture constructs. Whereas for both cell types similar staining for type II collagen was detected, in ACPC constructs distinctly weaker staining for undesired collagen I and only very low levels of alkaline phosphatase activity, a marker of terminal differentiation, were determined. In total, we provide further evidence that ACPCs can be regarded as a promising alternative cell source for cartilage engineering. Moreover, taken together with previous studies [21,44,45], the results underscore the importance of the choice of an appropriate hydrogel for each cell type. Agarose appeared to be a suitable hydrogel for ACPCs, and thus may also be utilized in future studies when further investigating layered zonal constructs, while other types of gels may be considered for layers in which MSCs are applied. In addition, direct co-cultures may be explored, also specifically investigating the effects of the hydrogel context.

## 4. Materials and Methods

### 4.1. Materials

Chemicals and cell culture reagents were purchased from Sigma-Aldrich (St. Louis, MO, USA) or Merck (Frankfurt, Germany) if not stated otherwise. Recombinant human FGF-basic/145aa (bFGF) and transforming growth factor-β 1 (TGF-β1) were obtained from BioLegend (London, UK). As primary antibodies, anticollagen VI antibody (ab6588), anticollagen I antibody (ab34710) and anticollagen II antibody (ab34712) (abcam, Cambridge, UK) were used. The secondary antibody was Alexa Fluor 488-conjugated AffiniPure goat antirabbit IgG (H+L) antibody (111-545-003) (Jackson Immuno Research, Ely, UK). Formaldehyde solution (37%), diaminobenzidine (DAB) and low melt agarose were purchased from Carl Roth (Karlsruhe, Germany), L–hydroxyproline from Merck (Darmstadt, Germany), ITS+ premix from Corning (Corning, NY, USA) and papain from Worthington (Lakewood, NJ, USA). Fetal bovine serum (FBS), MEM non-essential amino acids (NEAA) solution (100×), phosphate buffered saline (PBS), trypsin-EDTA (0.25%), penicillin-streptomycin (P/S; 100 U mL^−1^ penicillin, 0.1 mg mL^−1^ streptomycin) and calf intestinal alkaline phosphatase were obtained from Thermo Fisher (Waltham, MA, USA). QPCR master mix (Mesa Green qPCR Master Mix Plus for SYBR^®^ Assay No ROX) was purchased from Eurogentec (Seraing, Belgium). ImProm-II™ Reverse Transcription System was purchased from Promega (Madison, WI, USA) and proteinase K from Dako (Carpinteria, CA, USA). DAPI mounting medium ImmunoSelect^®^ was obtained from Dianova (Hamburg, Germany). Live/dead cell staining kit was purchased from PromoKine (Heidelberg, Germany).

### 4.2. Methods

#### 4.2.1. Cell Culture

Equine ACPCs and MSCs were harvested and characterized as was shown before [20] from two different donors (one for each cell type). In order to facilitate a direct comparison, the same donors were used as in a previous study [18]. Briefly, ACPCs and MSCs were respectively isolated from metacarpophalangeal joints and from bone marrow aspirates obtained from the sternum from recently deceased, skeletally mature horses (aged 3–10 years old), donated to science with informed and written consent by their owners, following the directives of the Institutional Animal Ethical Committee of Utrecht University. For the isolation of ACPCs, cartilage was cut with a scalpel under sterile conditions into 2 mm^2^ chips, then samples were digested at 37 °C for 2 h in 0.2% pronase and subsequently overnight in a 0.075% collagenase type II solution (Sigma-Aldrich, Zwijndrecht, The Netherlands). The obtained cells were plated on tissue culture dishes, which were previously coated with fibronectin (Sigma-Aldrich, Zwijndrecht, The Netherlands, 10 µg/mL in PBS, incubated for 1 h). After 20 min, non-attached cells were removed rinsing with PBS and the remaining cells were subcultured in expansion medium as detailed below. For MSC isolation, the mononuclear cell fraction from the bone marrow aspirates was obtained after centrifugation for 30 min at 300× *g* in a Ficoll–Paque density gradient (GE Healthcare, Zwijndrecht, The Netherlands), and cells were plated on tissue culture plastic for expansion. ACPCs and MSCs were used for three independent experiments, in which they were cultured in expansion medium up to passage 4. Medium for MSCs: DMEM high glucose 4.5 g L^−1^, supplemented with 10% FBS, 1 ng mL^−1^ bFGF, and 1% P/S; for ACPCs: DMEM high glucose 4.5 g L^−1^, supplemented with 10% FBS, 1% MEM NEAA, 0.2 mM L-ascorbic acid 2-phosphate, 5 ng mL^−1^ bFGF, and 1% P/S.

For 3D culture, 4% low melt agarose was prepared in PBS and kept at 37 °C. ACPCs or MSCs at passage 4 were suspended in the same volume of PBS and mixed 1:1 with the agarose, resulting in a 2% agarose cell suspension with 20.0 × 10^6^ cells mL^−1^. This cell suspension was cast in cylindrical silicone molds with a diameter of 6 mm and quickly cooled down to solidify the agarose gel. Monoculture constructs containing MSCs or ACPCs alone were prepared with 55 µL of cell suspension. For zonal constructs, 35 µL MSC suspensions were cast first, then 20 µL ACPC suspensions were cast on top of the MSC layer (Supplementary Figure S1). All day 1 samples were harvested after 24 h under normoxic conditions. The other constructs were cultured for four weeks in chondrogenic differentiation medium (DMEM high glucose 4.5 g L^−1^, supplemented with 1% *v/v* ITS+ premix, 0.2 mM L-ascorbic acid-2-phosphate, 100 nM dexamethasone, 10 ng mL^−1^ TGF-β1, 1% P/S). Each construct was cultured in 1 mL medium, which was refreshed every 2–3 days. Normoxic culture conditions were 21% oxygen and hypoxic culture conditions were 2% oxygen in the gas phase. A Binder CB150 CO_2_ incubator with O_2_ regulation (Binder, Neckarsulm, Germany) was used to create hypoxic conditions. For each of the three independent experiments, *n* = 3 biological replicates (constructs) were analyzed per group and representative results from one experiment were shown.

#### 4.2.2. Cell Viability Assay

The cell viability of agarose-encapsulated cells was determined using a live/dead cell staining kit (PromoKine, Heidelberg, Germany). Living cells were stained with calcein acetoxymethyl ester (calcein-AM) (green) and dead cells were stained with ethidium homodimer III (red). Hydrogels were washed with PBS and then incubated with calcein-AM and ethidium homodimer III for 45 min. After incubation, hydrogels were washed again in PBS before images were taken with a fluorescence microscope (Olympus BX51/DP71, Olympus, Hamburg, Germany).

#### 4.2.3. Quantitative Biochemical Analysis

Constructs (*n* = 3) were harvested after one, 14, and 28 days, and DNA, GAG, and hydroxyproline contents in the constructs were measured [61]. In brief, samples were homogenized using a TissueLyser (Qiagen, Hilden, Germany) at 25 Hz for 5 min. Then, they were digested in a papain solution (3 U mL^−1^) for 16 h at 60 °C. For fluorometric measurement of DNA content, Hoechst 33258 DNA intercalating dye (Ex: 360 nm, Em: 460 nm) was used with salmon testes DNA as standard [62]. Hydroxyproline content was quantified after acid hydrolysis and reaction with DAB and chloramine T at 560 nm, with L-hydroxyproline as the standard. The collagen content was determined by using a hydroxyproline-to-collagen ratio of 1:10 [63,64]. The amount of GAG was measured by DMMB assay at 525 nm, using bovine chondroitin sulfate as standard [65].

#### 4.2.4. Alkaline Phosphatase Activity Assay

Alkaline phosphatase (ALP) activity was investigated using a p-nitrophenyl phosphate (pnPP) phosphatase kinetic assay [66] after homogenization of the constructs (*n* = 3) with a TissueLyser (5 min, 25 Hz). SIGMAFAST™ p-nitrophenyl phosphate tablets (Merck, Frankfurt, Germany) were used to prepare a substrate solution. Dephosphorylation of pnPP by ALP resulted in the production of a yellow compound that was measured for 30 min at 405 nm using an Infinite M200 Pro plate reader (Tecan, Männedorf, Switzerland). One measurement was performed every two minutes with a shaking step in between. Readings at 655 nm were subtracted to correct for non-specific background values. Calf intestinal alkaline phosphatase was used as standard.

#### 4.2.5. Histology and Immunohistochemistry

Constructs were fixed in 3.7% PBS-buffered formalin overnight, then embedded in Tissuetek after dehydration, flash-frozen in liquid nitrogen, and cut into 6-µm-thick sections using a cryostat (Leica Biosystems, Wetzlar, Germany). For histological analysis of GAG deposition in the constructs, sections were stained with Weigert′s hematoxylin, fast green, and safranin-O [67]. In brief, sections were rinsed for 1 min in ddH_2_O and then incubated in Weigert’s hematoxylin solution for 6 min. Sections were rinsed in 0.5% hydrochloric acid in ethanol, then placed under running tap water for 5 min afterwards. They were incubated with 0.02% fast green solution for 4 min and then rinsed with 1% acetic acid. For 6 min, sections were incubated in 0.1% safranin–O solution, then they were washed with ethanol for 1 min, then with 2-propanol for 2 min, then with xylene for 2 min. Finally, samples were mounted using entellan. Deposited collagen was histologically assessed by staining of sections with Weigert’s hematoxylin for 8 min and picrosirius red for 60 min. Between these two steps, sections were put under running tap water for 10 min. After incubation with picrosirius red, sections were washed with 0.5% acetic acid for 5 min, then in ethanol for 1 min, in 2-propanol for 2 min, in xylene for 2 min, then finally mounted using entellan [68].

For immunohistochemical analysis of type I, II and VI collagen, sections were washed for 3 min in PBS, pretreated with proteinase K (10 min at 37 °C) for antigen retrieval, and then washed three times for 3 min with PBS. Afterwards, sections were blocked with 1% (*w/v*) BSA dissolved in PBS for 30 min and stained with the primary antibodies anticollagen I (1:200), anticollagen II (1:200) or anticollagen VI (1:200) overnight at 4 °C. On the next day, sections were washed three times for 3 min with PBS and then incubated with the secondary antibody for 60 min. As the secondary antibody, Alexa Fluor 488-conjugated AffiniPure goat antirabbit IgG (H+L) (1:400) was used. Afterwards, sections were mounted using the DAPI mounting medium ImmunoSelect^®^. Images were taken with a microscope BX51 with a DP71 camera (Olympus, Hamburg, Germany).

#### 4.2.6. RT-qPCR Analysis

Gene expression analysis was performed on day 1, day 14, and day 28 for chondrogenically differentiated agarose constructs. Harvested samples (*n* = 3) were homogenized with a TissueLyser (5 min, 25 Hz). Then, mRNA was isolated from homogenized samples using TRIzol reagent and cDNA synthesis was performed using the ImProm-II™ Reverse Transcription System. For amplification and detection of the target gene, the respective primers (Supplementary Table S2) and Mesa Green qPCR Master Mix Plus for SYBR^®^ Assay No ROX were utilized. The following cycling conditions were used: 95 °C for 6 min, then 40 cycles with 95 °C for 15 s, then 60 °C for 30 s, and 72 °C for 30 s. Relative expression levels of *ACAN*, *COL2A1*, *COL1A1*, and *PRG4* mRNA were determined by normalization to housekeeping gene *HPRT1* for each group and time point, and further to d1 of ACPCs using the 2^−ΔΔCt^ method. Relative expression levels of HIF target genes *PGK1, GLUT1,* and *CXCL12* at day 7 were determined by normalization to housekeeping gene *HPRT1* and further to the respective normoxia value.

#### 4.2.7. Statistical Analysis

The statistical significance of DNA, GAG, and collagen assays was assessed with Graph Pad Prism 6.0 (GraphPad Software, La Jolla, CA, USA) and a one-way ANOVA with a Tukey’s post-hoc test. The statistical significance of ALP assays and RT-qPCR were analyzed with Graph Pad Prism 6.0 (GraphPad Software, La Jolla, USA) and a two-way ANOVA with a Tukey’s post-hoc test. Statistical significance of HIF target genes was assessed with Graph Pad Prism 6.0 and Student’s *t*-test.

## Supplementary Materials

Supplementary materials can be found at https://www.mdpi.com/1422-0067/21/19/7071/s1. Figure S1: Schematic depiction of the structure of zonal (co-culture) and the non-zonal (monoculture) constructs. Figure S2: DNA content in ACPC, MSC, and zonal constructs under normoxic and hypoxic conditions. Figure S3: Live/dead staining. Figure S4: Staining of ACPC, MSC, and zonal constructs at d1 for type II and type I collagen. Figure S5: Staining of ACPC, MSC, and zonal constructs cultured under normoxic and hypoxic conditions for type VI collagen. Figure S6: Relative gene expression of HIF target genes in ACPC and MSC constructs. Table S1. Studies comparing ACPC chondrogenesis to other cell types in different hydrogels. Table S2. Primer sequences for RT-qPCR analysis.

## Abbreviations

*ACAN*	Aggrecan (name of gene)
ACPCs	Articular cartilage progenitor cells
ACI	Autologous chondrocyte implantation
ALP	Alkaline phosphatase
bFGF	Basic fibroblast growth factor
cDNA	Complementary deoxyribonucleic acid
*COL1A1*	Type I collagen α1-chain (name of gene)
*COL2A1*	Type II collagen α1-chain (name of gene)
*CXCL12*	C–X–C motif chemokine ligand 12
d1	Day 1
d14	Day 14
d28	Day 28
DAB	Diaminobenzidine
ddH_2_O	Double distilled water
DMEM	Dulbecco′s modified Eagle′s medium
DMMB	Dimethylmethylene Blue
DNA	Deoxyribonucleic acid
ECM	Extracellular matrix
EDTA	Ethylenediamine tetra-acetic acid
FBS	Fetal bovine serum
GAGs	Glycosaminoglycans
*GLUT1*	Glucose transporter 1
HA–SH/P(AGE–co–G)	thiol-functionalized hyaluronic acid/allyl-functionalized poly(glycidol)
HIF	Hypoxia inducible factor
HPRT1	Hypoxanthine Phosphoribosyl-transferase 1
Hz	Hertz
IgG	Immunoglobulin G
L^−1^	Per liter
MEM	Modified Eagle’s medium
mRNA	Messenger ribonucleic acid
MSCs	Mesenchymal stromal cells
NEAA	Non-essential amino acids
PBS	Phosphate-buffered saline
*PGK1*	Phosphoglycerate kinase 1
pnPP	p-nitrophenyl phosphate
*PRG4*	Proteoglycan 4
P/S	Penicillin–streptomycin
RT-qPCR	Reverse transcription quantitative polymerase chain reaction
TGF-β1	Transforming growth factor-β1
U	Unit
*v/v*	Volume per volume
