# Supplementary material for: Differential Production of Cartilage ECM in 3D Agarose Constructs by Equine Articular Cartilage Progenitor Cells and Mesenchymal Stromal Cells
Source: Int J Mol Sci. 2020 Sep 25;21(19):7071. doi: 10.3390/ijms21197071 (PMC7582568; doi:10.3390/ijms21197071)
Supplement: Supplementary file 1 [file ijms-21-07071-s001.pdf]

International Journal of
Molecular Sciences

MDPI

Supplementary Material to the Manuscript

Differential Production of Cartilage ECM in 3D Agarose Constructs by Equine Articular Cartilage Progenitor Cells and Mesenchymal Stromal Cells

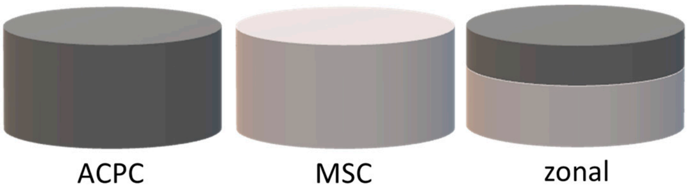

Supplementary Figure S1. Schematic depiction of the structure of zonal (co-culture) and the non-zonal (monoculture) constructs.

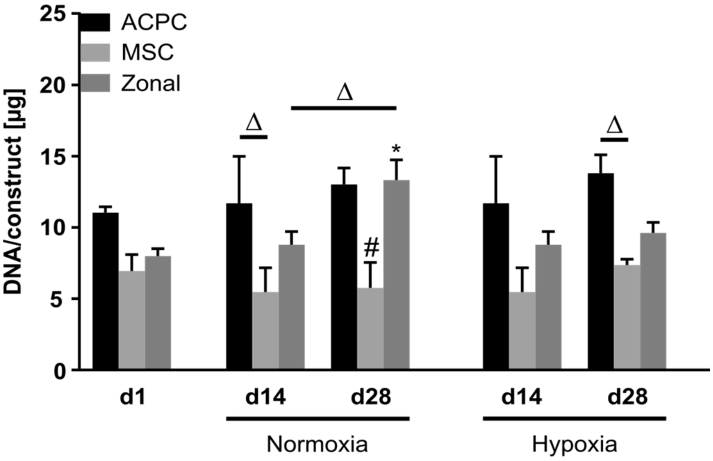

Supplementary Figure S2. DNA content in ACPC, MSC, and zonal constructs under normoxic and hypoxic conditions. Biochemical analysis of DNA amounts in agarose hydrogel constructs, seeded with 20.0 × 10^6 cells mL^{-1}, after 1, 14, and 28 days of chondrogenic differentiation under normoxic (21% O_2) or hypoxic (2% O_2) conditions. ACPCs and MSCs were either cultured alone or in zonally layered co-culture constructs. Data are presented as means + standard deviation (n = 3 biological replicates). Note: (*) indicates statistically significant differences between a d14 or d28 value and the corresponding d1 value of the same group ($p < 0.05$); (#) indicates statistically significant differences between this group and the other two groups that share the same time point and oxygen conditions ($p < 0.05$); (Δ) indicates statistically significant differences between groups, or within a group between time points ($p < 0.05$).

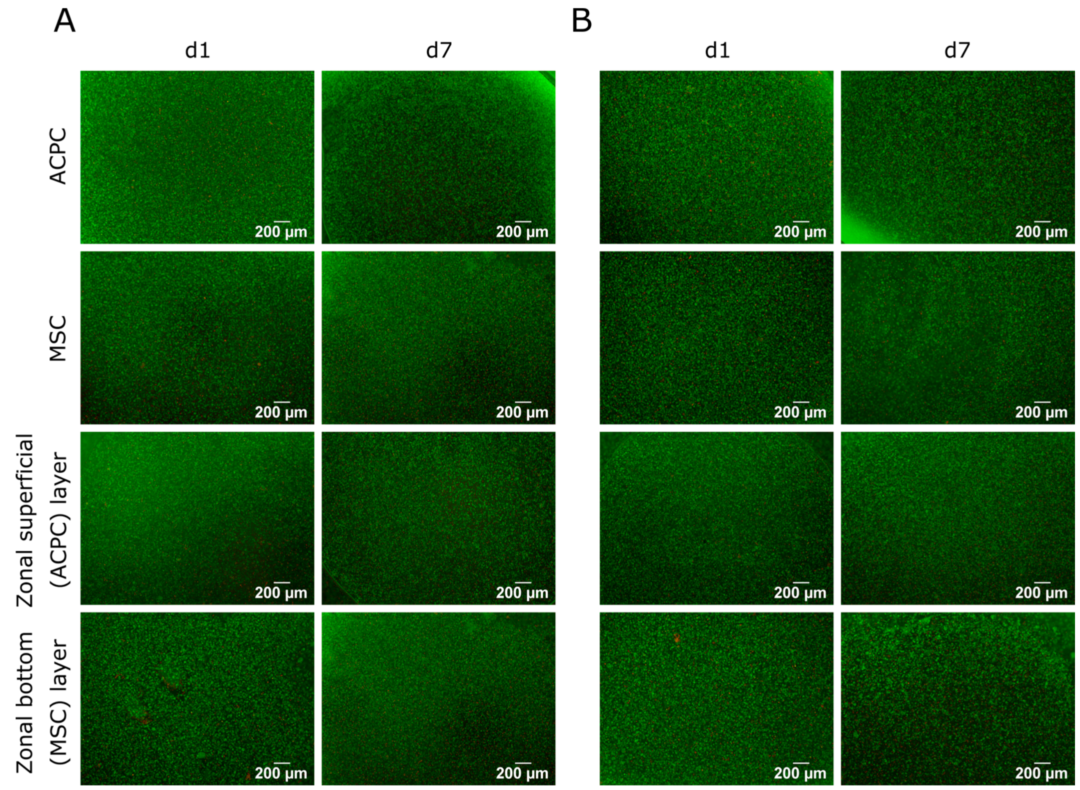

Supplementary Figure S3. Live/dead staining. Staining of live and dead cells in agarose hydrogel constructs, seeded with 20.0 × 10^6 cells mL^{-1}, after 1 and 7 days of chondrogenic differentiation under normoxic and hypoxic conditions. ACPCs and MSCs were either cultured alone or in zonally layered co-culture constructs. (**A**) Staining of live (green) and dead (red) cells on day 1 and day 7 under normoxic conditions. (**B**) Staining of live (green) and dead (red) cells on day 1 and day 7 under hypoxic conditions.

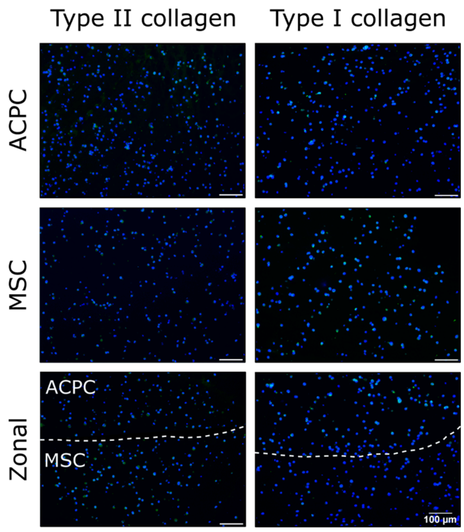

Supplementary Figure S4. Staining of ACPC, MSC, and zonal constructs at d1 for type II and type I collagen. Immunohistochemical staining for type II and type I collagen in agarose hydrogel constructs, seeded with 20.0 × 10^6 cells mL^{-1}, after 1 day of chondrogenic differentiation. ACPCs and MSCs were either cultured alone or in zonally layered co-culture constructs. In zonal constructs, the upper layer contained ACPCs and the lower layer contained MSCs (indicated by the dashed line).

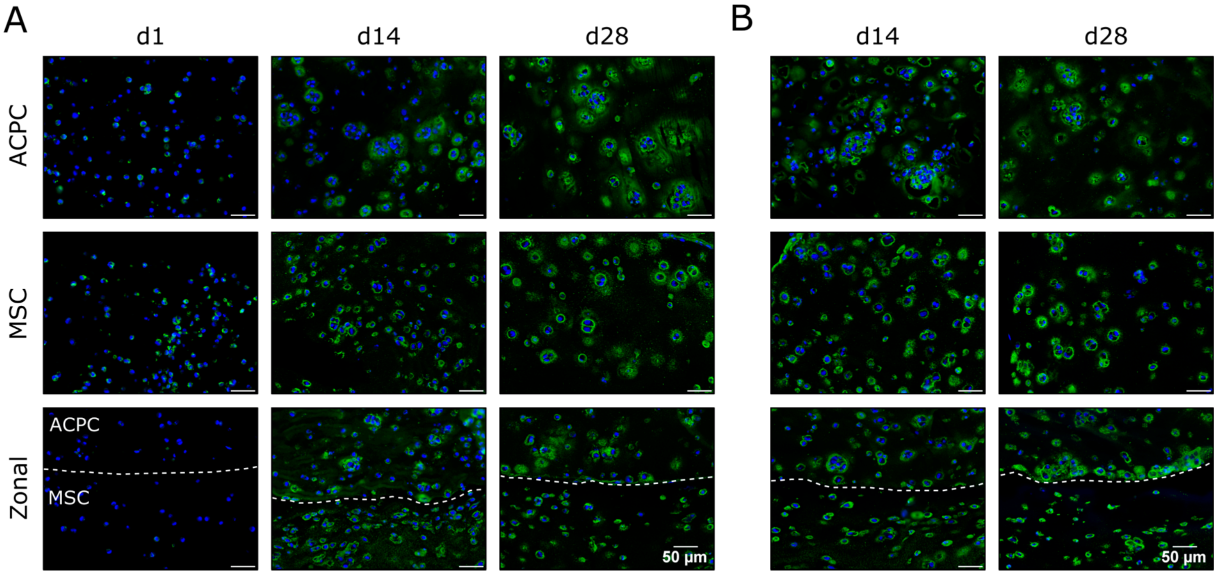

Supplementary Figure S5. Staining of ACPC, MSC, and zonal constructs cultured under normoxic and hypoxic conditions for type VI collagen. Immunohistochemical staining for type VI collagen in agarose hydrogel constructs, seeded with 20.0 × 10^6 cells mL^{-1}, after 1, 14, and 28 days of chondrogenic differentiation. ACPCs and MSCs were either cultured alone or in zonally layered co-culture constructs. (**A**) Immunohistochemical staining for type VI collagen after normoxic culture conditions. (**B**) Immunohistochemical staining for type VI collagen after hypoxic culture conditions. In zonal constructs, the upper layer contained ACPCs and the lower layer contained MSCs (indicated by the dashed line).

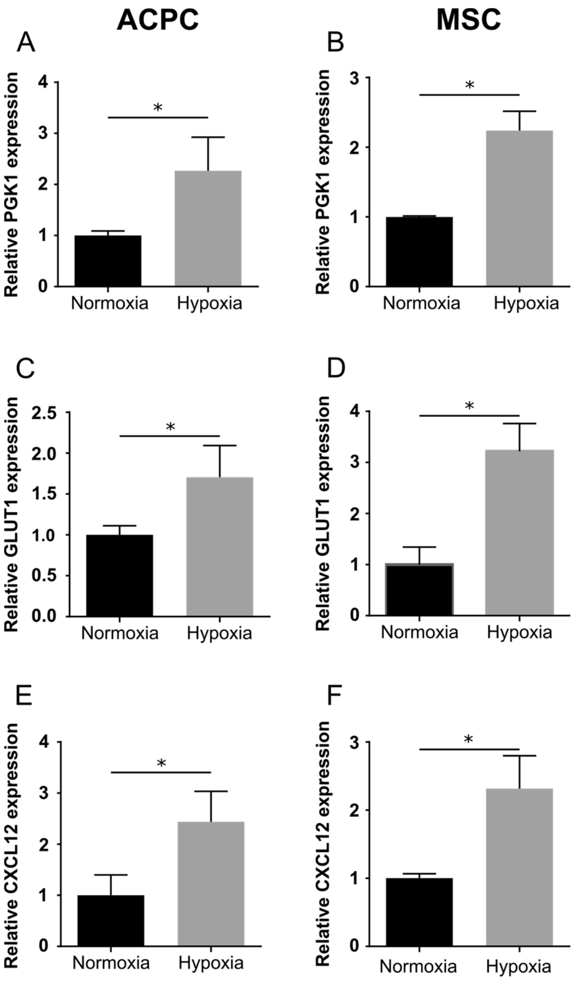

Supplementary Figure S6: Relative gene expression of HIF target genes in ACPC and MSC constructs. Gene expression as determined by RT-qPCR in agarose hydrogel constructs, seeded with 20.0×10^6 cells mL^{-1}, after 7 days of chondrogenic differentiation under hypoxic and normoxic conditions. Relative expression of *PGK1* in (**A**) ACPC constructs and (**B**) MSC constructs. Relative expression of *GLUT1* in (**C**) ACPC constructs and (**D**) MSC constructs. Relative expression of *CXCL12* in (**E**) ACPC constructs and (**F**) MSC constructs. Data are presented as means ± standard deviations ($n = 3$ biological replicates). Note: (*) indicates statistically significant differences between two values ($p < 0.05$).

Supplementary Table S1. Studies comparing ACPC chondrogenesis to other cell types in different hydrogels.

Study	Hydrogel	Cells	ECM Production
Levato et al. *Acta Biomater.*, 2017 [20]	gelMA	ACPCs, MSCs, chondrocytes	GAG: MSCs > ACPCs > chondrocytes<br>Type II collagen: MSCs > ACPCs > chondrocytes
Mouser et al. *Connect. Tissue Res.*, 2020 [21]	gelMA, gelMA/gellan, gelMA/gellan/HAMA	ACPCs, MSCs, chondrocytes	GAG: MSCs > ACPCs > chondrocytes<br>Type II collagen: MSCs > ACPCs > chondrocytes
Mancini et al. *Biofabrication*, 2020 [18]	HA–SH/P(AGE–co–G)	ACPCs, MSCs	GAG: MSCs > ACPCs<br>Type II collagen: MSCs > ACPCs
Present study	Agarose	ACPCs<br>MSCs	GAG: ACPC > MSCs<br>Type II collagen: ACPCs ≈ MSCs

Supplementary Table S2. Primer sequences for RT-qPCR analysis.

Target Gene	Primer Sequence (5′-3′)
ACAN	F: aatgggaaccagcctacacg
	R: gctctcct tg tgctgcact
COL2A1	F: acctcgtggcagagatgga
	R: tgggcagcaaagtttccac
COL1A1	F: agggtgagacaggcgaaca
	R: gggaccttgttcaccaggag
HPRT1	F: aagcttgctggtgaaaag[1]
	R: gcatatcctacgacaaact[1]
PRG4	F: cttcccatttacttgttgctg[1]
	R: tagaatacccttccccacat[1]
PGK1	F: ggaagaagggaagggcaaag
	R: ggaaagtgaagctcggaaggt
CXCL12	F: gccagagccaacatcaaac
	R: tcagtttcgggtcaatgcac
GLUT1	F: ccctgcaccagttgagtgtc
	R: gggaggaaggtgatgctcag

[1] Primer sequences were adopted from [20].
